# Disaccharides obtained from carrageenans as potential antitumor agents

**DOI:** 10.1038/s41598-019-43238-y

**Published:** 2019-04-30

**Authors:** Gustavo H. Calvo, Vanina A. Cosenza, Daniel A. Sáenz, Diego A. Navarro, Carlos A. Stortz, Mariela A. Céspedes, Leandro A. Mamone, Adriana G. Casas, Gabriela M. Di Venosa

**Affiliations:** 1 Centro de Investigaciones sobre Porfirinas y Porfirias (CIPYP), Hospital de Clínicas José de San Martin, Universidad de Buenos Aires and CONICET, Av. Córdoba 2351, 1er subsuelo, Ciudad Autónoma de Buenos Aires, 1120AAF Argentina; 2 CIHIDECAR- CONICET - Departamento de Química Orgánica, Facultad de Ciencias Exactas y Naturales, Universidad de Buenos Aires, Ciudad Universitaria, Pab. 2, Ciudad de Buenos Aires, 1428 Argentina

**Keywords:** Drug development, Cell biology

## Abstract

Carrageenans are sulfated galactans found in certain red seaweeds with proven biological activities. In this work, we have prepared purified native and degraded κ-, ι-; and λ-carrageenans, including the disaccharides (carrabioses) and disaccharide-alditols (carrabiitols) from seaweed extracts as potential antitumor compounds and identified the active principle of the cytotoxic and potential antitumor properties of these compounds. Both κ and ι-carrageenan, as well as carrageenan oligosaccharides showed cytotoxic effect over LM2 tumor cells. Characterized disaccharides (carrabioses) and the reduced product carrabiitols, were also tested. Only carrabioses were cytotoxic, and among them, κ-carrabiose was the most effective, showing high cytotoxic properties, killing the cells through an apoptotic pathway. In addition, the cells surviving treatment with κ-carrabiose, showed a decreased metastatic ability *in vitro*, together with a decreased cell-cell and cell-matrix interactions, thus suggesting possible antitumor potential. Overall, our results indicate that most cytotoxic compounds derived from carrageenans have lower molecular weights and sulfate content. Potential applications of the results emerging from the present work include the use of disaccharide units such as carrabioses coupled to antineoplasics in order to improve its cytotoxicity and antimetastatic properties, and the use of ι-carrageenan as adjuvant or carrier in anticancer treatments.

## Introduction

For decades, red seaweeds have been an important source of hydrocolloids (i.e. carrageenans and agarans), widely used both in food and non-food industries^1^. Carrageenans, in particular, are sulfated galactans with a backbone of [ → 3)-β-d-Galp-(1 → 4)-α-d-Galp-(1 → ] and differ from each other in monosaccharide composition, degree and positions of sulfate groups, possible replacement of the α-galactose unit (G) by a 3,6-anhydrogalactose moiety (A) and molecular weights. Classification of these galactans depends on the presence or absence of the anhydro sugar and the position of the sulfate groups.

Three carrageenan types, so-called κ-, ι- and λ-carrageenans, are of commercial significance. These carrageenans are attractive due to their rheological properties as stabilizers and thickening or gelling agents^2^. Both κ- and ι-carrageenans can form gels upon cooling or in the presence of K^+^ or Ca^2+^ counterions^3^, whereas λ-carrageenan hardly gels, or needs high concentrations of K^+^ to give weak gels^4,5^. Thus, it is used as a thickening agent^6^. They can be used in the food, pharmaceutical, cosmetics, printing and textile industries. In addition, it was reported their use as renewable, ecological and nontoxic mobility control agents to increase sweep efficiency for oil recovery in dwindling petroleum reserves^7^.

Besides their commercial interest, carrageenans are also known to have biological properties as well as low toxicity. These properties are originated in their negative charge and their resemblance to the glycosaminoglycans (GAGs) present in the cell membranes of mammals^8^. The antiviral activity is the most studied biological property and it is attributed to the capacity of the sulfated polysaccharides to hinder the viral entry to the host^9^. Several reports have shown the effectiveness of carrageenans against different enveloped and non-enveloped viruses^9^. Actually, it was observed that high molecular weight and high degrees of sulfation favor the antiviral activity^8^.

The antitumor activity of carrageenans has been less explored. Nonetheless, it is an area of great potential^10^. The antitumor activity of these polysaccharides could be related to the destabilization of the interaction of the GAG portion of the proteoglycans and the extracellular matrix proteins, thus eliminating the adhesion of cancer cells to matrices, which is necessary in metastasis spread^11^.

λ-Carrageenan from *Chondrus ocellatus*^12^, κ-carrageenan from *Kappaphycus alvarezii*^13^ as well as commercial κ- and λ-carrageenan^14,15^ showed antitumor activity *in vivo* and/or cytotoxic activity *in vitro*. They possess either a direct inhibitory action on cancer cells and tumors or influence different stages of carcinogenesis and tumor development, recover the broken balance between proliferation and programmed cell death (apoptosis) and are useful for cancer prevention^16^. Actually, the polysaccharide isolated from *C*. *ocellatus* was only active *in vivo*, probably related to immunomodulating activity of the galactan. Due to its unique secondary and tertiary structure, carrageenan is resistant to biochemical degradation by lysosomal glycosidases. Carrageenan containing phagolysosomes eventually rupture due to osmotic swelling. The consequent release of hydrolytic enzymes into the cytosol causes irreversible damage and eventual lysis of macrophages^17^.

Taking into consideration the scarcity of structural analysis performed to the antitumor agents^12,13,18^, few correlations have been observed between the chemical structure and the antitumor activity. Nonetheless, it was observed that depolymerization of carrageenans (mainly κ-, ι- and λ-carrageenan) favors their antitumor activity^11,12,18–21^. Actually, when oligosaccharides of different molecular weights were evaluated, those with the lowest values showed higher activity^12,18,21^. Interestingly, there are no reports of the antitumor activity of the isolated disaccharidic units, i.e. carrabiose or neocarrabiose.

In most cases, anticancer activity was only observed *in vivo*^11,12,19^ though *in vitro* cytotoxicity of degraded κ- and ι-carrageenan was also observed^18,21^.

Nowadays, almost 75% of the anticancer agents used in chemotherapy are derived from natural sources, and plants are an important source of new promising therapies^22^.

As carbohydrates are involved in tumor development, progression, invasion and metastasis, glycan structural abnormalities were always considered as golden bio-marks to diagnose and rank cancer status^23^, so carbohydrates including mono- or disaccharides were incorporated to non-specific antitumor molecules in order to improve the targeting and efficacy properties. The disaccharide moiety of bleomycin (BLM), composed of 3-*O*-carbamoyl-D-mannose and L-gulose, was employed to improve targeting of tumor cells is one of the recently employed strategies^24^.

In addition, research on marine polysaccharide-based nanomaterials is emerging in nanobiotechnological fields such as drug delivery^25^. Both chitosan and carrageenan are marine-derived polymers which have demonstrated to assemble into nanoparticles^26^, and κ- and λ- (but not ι-carrageenans) have been employed as carriers of anticancer drugs^27^ and as adjuvants on the preparation of dendritic based vaccines for cancer treatment^15^.

**Figure 1:**
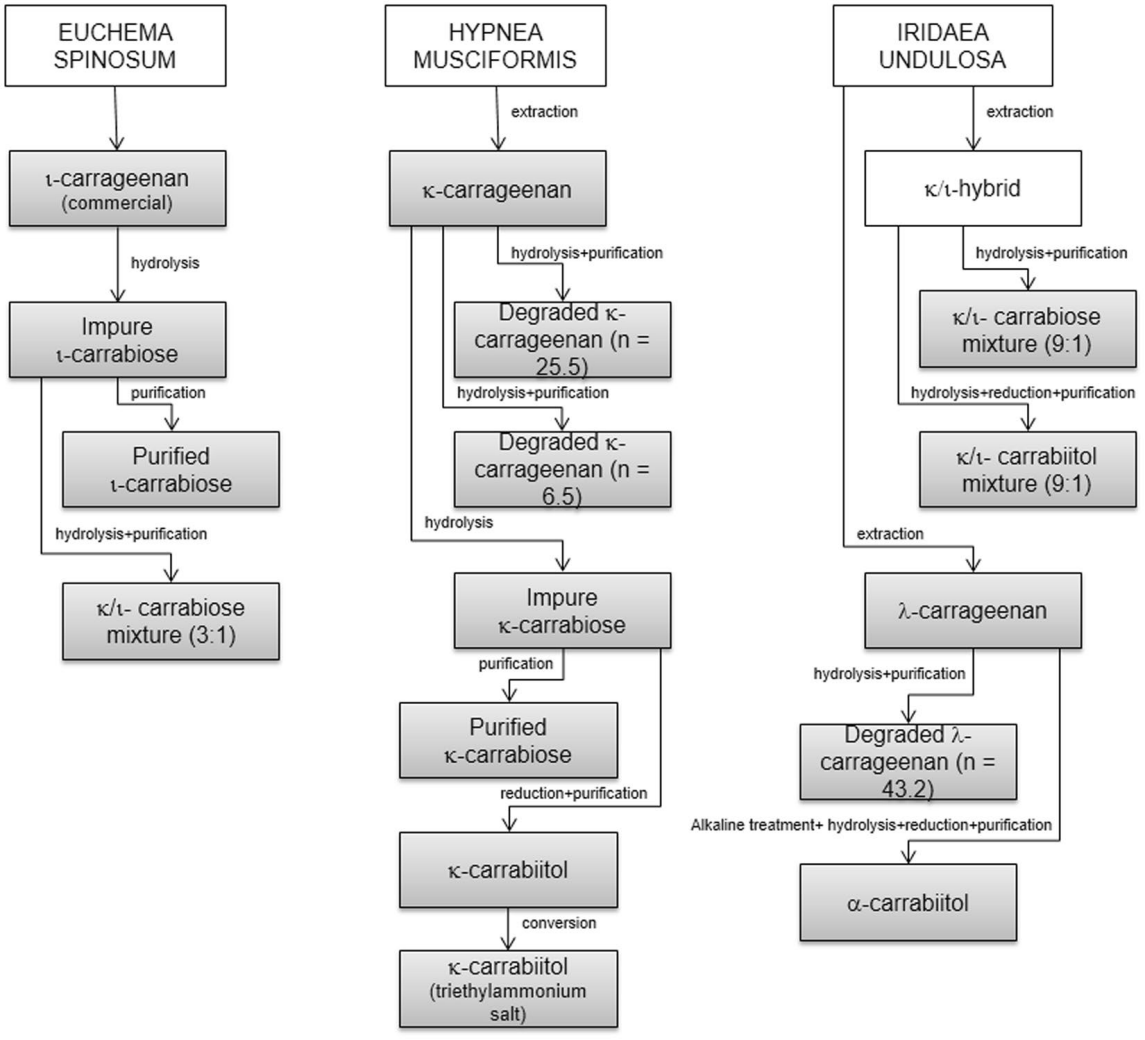
Pathway for the preparation of the different compounds. In gray, the compounds assessed.

The aim of the present work, was to study purified native and degraded carrageenans, and their disaccharides, obtained from extracts from *Hypnea musciformis*, *Iridaea undulosa* and *Euchema spinosum* (Fig. 1) as potential cytotoxic and antitumor compounds.

## Materials and Methods

### Chemicals

**Figure 2:**
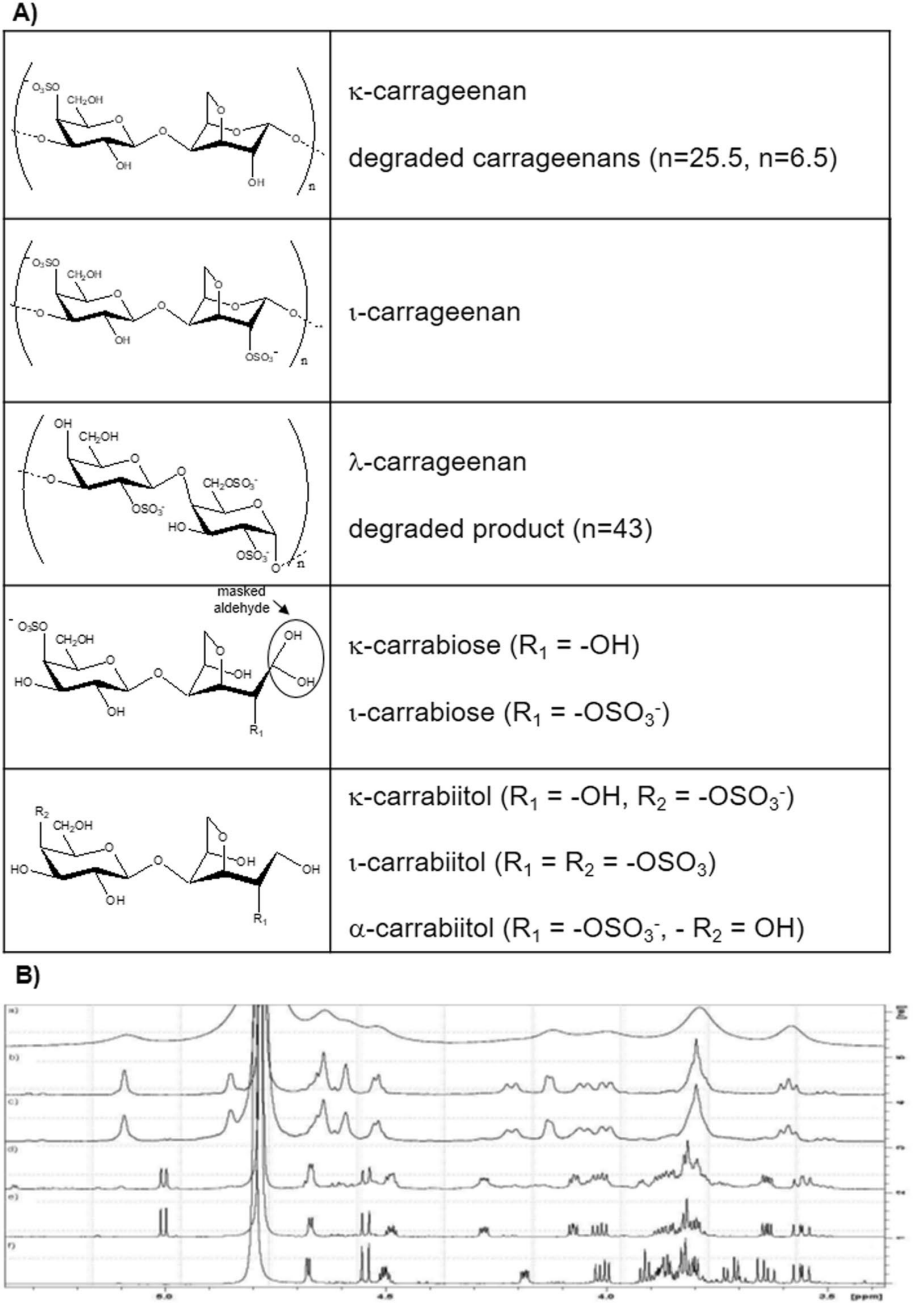
Polysaccharides, oligosaccharides and disaccharides: structures and characterization. (**A**) Chemical structures of κ-carrageenan and its degraded product, ι-carrageenan, λ-carrageenan and its degraded product, κ-carrabiose, ι-carrabiose, κ-carrabitol, ι-carrabiitol and α-carrabiitol. (**B**) 500 MHz ^1^H-NMR spectra of κ-carrageenan and its oligosaccharides in D_2_O: (a) undegraded κ-carrageenan, (b) degraded κ-carrageenan (n = 25.5), (c) degraded κ-carrageenan (n = 6.5), (d) impure κ-carrabiose, (e) κ-carrabiose, and f) κ-carrabiitol.

3-[4,5-dimethylthiazol-2-yl]-2,5-diphenyltetrazoliumbromide (MTT) and ι-carrageenan (Fig. 2A) from *Euchema spinosum* (460 kDa) were obtained from Sigma-Aldrich (Poole, UK). The rest of the chemicals employed were of analytical grade.

### Material

Samples of *Hypnea musciformis* (Wulfen) Lamoroux Foslie were collected near Natal (Rio Grande do Norte), Brazil (5°37′52′′S, 35°13′03′′W). Samples of *Iridaea undulosa* were collected near Puerto Madryn (Province of Chubut), Argentina, and sorted at the Centro National Patagónico (Puerto Madryn, Chubut). The seaweeds were sorted, air dried, cleaned manually and milled to a fine powder before extraction.

### Extraction and fractionation

The extraction of the carrageenans from *Hypnea muscifomis* and KCl fractionation procedures were described elsewhere^6^. A κ-carrageenan (215 kDa) (Fig. 2A,Ba) was obtained after precipitating the polysaccharides extracted in hot water with 0.125 M KCl, dialyzing and lyophilizing.

The extraction and fractionation of the carrageenans from *Iridaea undulosa* (both tetrasporic and cystocarpic) were also described elsewhere^5^. Extraction at room temperature of the cystocarpic samples gave a κ/ι/μ/ν-hybrid which, after alkaline treatment, yielded a κ/ι-hybrid carrageenan^28^. Room-temperature extraction of the tetrasporic plant afforded a λ-carrageenan (619 kDa) which was further purified by precipitation with KCl (fraction precipitated between 1 M and 1.25 M, Fig. 2A).

### Generation of oligosaccharides from κ-carrageenan

#### Oligosaccharide with n = 25.5

One hundred mg of κ-carrageenan from *H*. *musciformis* were dissolved in 5 ml of water and eluted through an Amberlite IR-120 column (H^+^ form) in order to obtain the acid form. Thirty ml were collected in an ice bath. The acid solution was heated at 60 °C for 6 h^29^. The solution was cooled, neutralized using NaHCO_3_ (ss), dialyzed (cut-off 1,000) and freeze-dried, yielding the corresponding oligosaccharide with a mean number of disaccharidic units (n) = 25.5 (determined from its molecular weight of 10.4 kDa, Fig. 2A,Bb).

#### Oligosaccharide with n = 6.5

One hundred mg of the κ-carrageenan were treated with 25 ml 0.1 M trifluoroacetic acid (TFA) at 80 °C for 0.5 h. The solution was neutralized using NaHCO_3_ (ss), dialyzed (cut-off 1,000) and freeze-dried, yielding an oligosaccharide with a mean number of disaccharidic units (n) = 6.5 (determined by ^1^H-NMR, Fig. 2A,Bc).

### Generation of an oligosaccharide from λ-carrageenan

One hundred mg of λ-carrageenan from *Iridaea undulosa* were treated with 25 ml of 0.1 M TFA at 80 °C for 3 h. Then, the solution was neutralized using NaHCO_3_ (ss), dialyzed (cut-off 3,500) and freeze-dried obtaining an oligosaccharide with a mean number of disaccharide units (n) = 43.2 (determined from its molecular weight of 27.2 kDa, Fig. 2A).

### Generation of κ-carrabiose

One hundred mg of κ-carrageenan from *H*. *musciformis* were treated with 25 ml of 0.1 M TFA at 80 °C for 3 h. The solution was evaporated at reduced pressure. The solid stuck into the vessel walls was redissolved in water and reevaporated, repeating the procedure three times in order to eliminate the TFA. Finally, the hydrolysis product was dissolved in water and freeze dried affording an impure κ-carrabiose (minor presence of oligosacharides with n ≥ 2 and monosaccharides was confirmed by NMR, representing less than 20% of the product, Fig. 2Bd).

The impure product was further purified by gel permeation chromatography using Biogel P2 as stationary phase (on a column of 30 cm × 1.5 cm), eluted with plain water. Glucose and blue dextran were used as markers of the V_f_ and V_0_ respectively. The sample (30 mg) was applied in 1 ml of distilled water, and fractions of 2 ml were collected and analyzed using the phenol-H_2_SO_4_ method^30^. Fractions corresponding to the major peak were pooled and freeze-dried yielding pure κ-carrabiose (Fig. 2A). ^1^H-NMR signals (500 MHz, Fig. 2Be): δ = 5.00 (A-1), 4.67 (G-4), 4.54 (G-1), 4.49 (A-5), 4.28 (A-4), 4.08 (A-3), 4.02 (A-6), 3.87 (G-5), 3.85 (A-6′), 3.82 (G-6,6′), 3.81 (G-3), 3.64 (A-2), 3.56 (G-2). ^13^C-NMR signals (125 MHz): 104.0 (G-1), 90.7 (A-1), 87.9 (A-4), 83.3 (A-3), 77.3 (G-4), 76.2 (A-5), 75.4 (G-5), 73.7 (A-2), 73.6 (A-6), 72.4 (G-3), 71.6 (G-2), 61.8 (G-6).

### Generation of ι-carrabiose

ι-Carrabiose (Fig. 2A) was generated and purified using the same procedure depicted for κ-carrabiose (section 2.6) starting from a commercial ι-carrageenan. During the purification step, two fractions were isolated: a major one composed mainly of ι-carrabiose (Fig. 2A) and a minor one composed of a mixture of κ- and ι-carrabiose in 3:1 ratio (as determined by ^1^H-NMR). More than one purification step was necessary for isolating pure ι-carrabiose. ^1^H-NMR signals for purified ι-carrabiose (500 MHz): 5.26 (A-1), 4.67 (G-4), 4.57 (G-1), 4.47 (A-5), 4.44 (A-4), 4.42 (A-2), 4.19 (A-3), 4.02 (A-6), 3.86 (G-5), 3.83 (A-6′), 3.81 (G-3), 3.80 (G-6,6′), 3.55 (G-2). ^13^C-NMR signals (125 MHz): 104.0 (G-1), 89.8 (A-1), 87.6 (A-4), 81.6 (A-3), 79.5 (A-2), 77.3 (G-4), 76.3 (A-5), 75.4 (G-5), 73.4 (A-6), 72.4 (G-3) 71.7 (G-2), 61.7 (G-6).

### Generation of a κ/ι-carrabiose mixture

Starting from the alkali treated κ/ι-hybrid carrageenan from cystocarpic *Iridaea undulosa*, and following the procedure stated in Section 2.6, a mixture of κ- and ι-carrabiose was obtained in a 9:1 ratio (as determined by ^1^H-NMR, Fig. 2A).

### Generation of κ-carrabiitol and its triethylamonium salt

Impure κ-carrabiose (20 mg) was treated with 5 mg NaBH_4_ and 10 ml of 1 M NH_4_OH. The reaction was left overnight. Then, the ammonia was evaporated at reduced pressure. The solid was redissolved in water and reevaporated three times. Finally, the solid was dissolved in 1 ml of water and purified as described in Section 2.6, obtaining κ-carrabiitol (Fig. 2A). ^1^H-NMR signals (500 MHz, Fig. 2Bf): 4.67 (G-4), 4.54 (G-1), 4.50 (A-5), 4.18 (A-4), 4.00 (A-6), 3.91 (A-3), 3.87 (G-5), 3.87 (A-2), 3.86 (A-6′), 3.81 (G-6,6′), 3.79 (G-3), 3.70 (A-1), 3.64 (A-1′), 3.55 (G-2). ^13^C-MNR signals (125 MHz): 104.1 (G-1), 88.3 (A-4), 84.1 (A-3), 77.3 (G-4), 76.5 (A-5), 75.4 (G-5), 72.4 (G-3), 72.1 (A-2), 73.5 (A-6), 71.6 (G-2), 63.5 (A-1), 61.8 ppm (G-6).

The product was then converted into its triethylamonium salt eluting 10 mg of the carrabiitol (dissolved in 1 ml) through an Amberlite IR-120 resin (Et_3_NH^+^ form). The salt was obtained by freeze-drying.

### Generation of a κ/ι-carrabiitol mixture

Using the protocol described in section 2.9, and the carrabiose mixture described in section 2.8, a κ-/ι-carrabiitol mixture (ratio 9:1) was obtained.

### Generation of α-carrabiitol

α-Carrabiitol (Fig. 2A, determined by ^1^H-NMR) was generated as described for production of κ-carrabiitol (Section 2.6 and 2.9) starting from the alkali treated product of λ-carrageenan, carried out as reported previously for the κ/ι-hybrid from *I*. *undulosa* (Section 2.2). ^1^H-NMR signals (500 MHz): 4.55 (A-2), 4.53 (G-1), 4.49 (A-5), 4.34 (A-4), 4.12 (A-3), 4.02 (A-6), 3.92 (G-4), 3.87 (A-1,1′), 3.84 (A-6′), 3.76 (G-6,6′), 3.74 (G-5), 3.67 (G-3), 3.53 (G-2). ^13^C-NMR signals (125 MHz): 104.0 (G-1), 87.1 (A-4), 82.4 (A-3), 78.6 (A-2), 76,7 (A-5), 76.1 (G-5), 73.5 (A-6), 73.4 (G-3), 71.6 (G-2), 69.4 (G-4), 61.8 (G-6), 61.3 (A-1).

### Gel permeation chromatography

GPC analysis was performed with a high performance liquid chromatography system equipped with a Shimadzu refractive index detector (Model RID-10A), and a Shimadzu LC-20AT pump. Polysaccharides were analyzed using two columns connected in series: a Supelco Progel-TKS G4000 and a Waters Ultrahydrogel 250 (300 × 7.8 mm). A degassed solution of 0.05 M NaNO_3_ prepared with ultra-pure water containing 0.02% NaN_3_ was used as solvent and eluent. One hundred μl of polysaccharide solutions (5 mg/ml) were filtered through a 0.22 μm PVDF membrane (GV, Millipore) and injected into the column using a manual valve. The eluent flow rate was of 0.6 ml/min and the temperature was held at 25 °C. The column was calibrated using dextran standards of different molecular weights (Sigma Aldrich, USA).

### Nuclear magnetic resonance (NMR)

The NMR spectra to analyze the structures and the composition of each mixture, were obtained on a Bruker Avance II 500 spectrometer at 500.13 (^1^H) and 125.77 (^13^C) MHz provided with a 5-mm probe, at room temperature, using ca. 20 mg polysaccharide in 0.7 ml of D_2_O. Acetone was added as internal standard (referred to Me_4_Si by calibrating the acetone methyl group to 31.1 ppm in ^13^C, 2.22 ppm in ^1^H). ^1^H NMR spectra were carried out with an acquisition time of 4.36 s, a pulse angle of 30°, and a pulse delay of 1 s.

### Cell lines

LM2 cell line derived from the murine mammary adenocarcinoma, M2^31^, was obtained from Instituto Roffo, Buenos Aires, Argentina. A panel composed of the following lines was also used: IGROV-1: human ovarian cancer; K562: Myelogenous leukemia cell line of the erythroleukemia type; B16-F10: mouse melanoma; MB49: mouse bladder cancer; A549: human bronchioloalveolar lung carcinoma and PAM212: murine cutaneous squamous cell carcinoma.

The cell lines (except for LM2) were obtained from the American Type Culture Collection (ATCC), USA. All the cells were maintained in RPMI-1640 medium supplemented with 5% serum for LM2 and 10% for the rest of the cells, 80 μg gentamycin/ml and 2 mM L-glutamine and kept in humidified atmosphere of 5% CO2 at 37 °C.

### MTT viability assay

Cell viability was assessed by the MTT assay^32^, which is a method based on the activity of mitochondrial dehydrogenases. Following appropriate treatments, a MTT solution was added to each well in a concentration of 0.5 mg/ml, and plates were incubated at 37 °C for 1 h. The resulting formazan crystals were dissolved in dimethyl sulfoxide and the absorbance was read at 560 nm.

The concentrations required to inhibit cell growth by 50% (IC_50_) were calculated from the abscissa intercept from logistic curves constructed by plotting cell survival (%) versus drug concentration (mg/ml).

### Determination of κ-carrabiose cellular uptake

With the aim of determining the likelihood of the involvement of active transport on the incorporation of κ-carrabiose to the cells, LM2 cells were exposed to κ*-*carrabiose (2 mg/ml) either at 4 °C or 37 °C during 90 min, time at which no cytotoxicity is induced by low temperature exposure^33^. Afterwards, MTT assay was routinely performed as described in 2.15.

### Cell cycle distribution

Cells (7 × 10^4^ cells/ml) were seeded in a 12 well plate, 24 h later κ-carrabiose was added and another 48 h later, the cells were tripsinized and centrifuged with the conditioned media, and the pellet was resuspended with 70% ethanol to fix the cells. Cells were exposed to ethanol at 4 °C for 30 min and washed in 2% bovine serum albumin in phosphated buffered saline. The pellets were incubated with 0.3 ml of 100 µg/ml RNAase during 30 min and then 50 µl of 1 mg/ml propidium iodide was added to stain DNA just before the cytometric analysis. The cell-cycle phase distribution was determined using a BD FACSCalibur flow cytometer (Becton Dickinson, San Jose, CA). The percentages of cells in each cell-cycle phase were analyzed using FlowJo vX software. Toxicity was checked by MTT viability assay in parallel experiments.

### Immunofluorescence assays for expression of cell-cell adhesion and cell-substrate adhesion proteins

To visualize the expression of the cell-cell adhesion proteins E-cadherin and β-catenin and the cell-matrix adhesion protein vinculin, immunofluorescence assays were carried out. LM2 cells (1 × 10^5^ cells/ml) grown on coverslips were fixed in 4% paraformaldehyde for 15 min. Samples were rehydrated in PBS, permeabilized with 1% Triton X-100 in PBS for 10 min and incubated with the anti E-cadherin, β-catenin or vinculin primary antibody for 1 h at 37 °C, washed with PBS, incubated with a FITC-labelled secondary antibody (1:500 in 0.1% BSA) and finally washed with PBS. The preparations were mounted in ProLong Gold antifade reagent (Invitrogen, USA). Microscopic observation and photography were performed with an Olympus photomicroscope BX51, equipped with a HBO 100 W mercury lamp and the green filter sets for fluorescence microscopy (545 nm, exciting filter BP 545).

### *In vitro* migration assay

Cell migration was determined using a wound healing assay. An amount of 2 × 10^5^ cells were plated in complete medium containing 10% fetal bovine serum into 6-multiwell plates and allowed to grow until confluence, time at which the cultures were wounded through the central axis with a sterile 200 μl tip. Cells were washed with PBS, refreshed with serum containing medium and incubated with different concentrations of κ-carrabiose. After overnight incubation (21 h) at 37 °C cells were fixed, stained with Crystal violet and photographed. Quantification of cell motility was performed by measuring the distance between the invading fronts of cells in six random selected microscopic fields for each condition. The degree of motility was expressed as a migration index (MI = (0 h wound − 24 h wound)/0 h wound). Images were acquired with an Olympus microscope with 10X objective and processed using image processing software.

### Statistical analysis

Values were expressed as mean ± standard deviations of the mean. Analysis of variance (ANOVA) with post hoc comparisons was used to determine statistical significance employing the raw non-relativized data (Graphpad Instat© software).

## Results and Discussion

### Analysis of cytotoxicity

In the present work we selected a tumor cell line for this preliminar cytotoxicity screening and to gain insight into the use of carrageenans as potential antitumor agents. Since the use of normal cell populations as controls proved not to be feasible on the screening of anticancer drugs^34^ we did not attempt to carry out any comparison between normal and tumor cell lines.

#### Polysaccharides

There are three main types of carrageenans, differentiated by the position and degree of sulfation and the presence or absence of 3,6-anhydrogalactose. κ-Carrageenan is composed of alternating 3-linked β-d-galactopyranose 4-sulfate and 4-linked 3,6-anhydro-α-d-galactopyranose units, thus having one negative charge per disaccharide repeating unit (Fig. 2A); ι-carrageenan has the same backbone but an additional sulfate group at C-2 of the α- unit (Fig. 2A), whereas λ-carrageenan has no 3,6-anhydrogalactose and carries three sulfate groups per disaccharidic unit (at C-2 of the β- unit and at C-2 and C-6 of the α- unit, Fig. 2C). The actual polysaccharides show small deviations from the ideal structures depicted in the Figures.

With the aim to establish the cytotoxicity of carrageenans, we exposed LM2 tumor cells to the different carrageenans and assessed cell viability.

**Figure 3:**
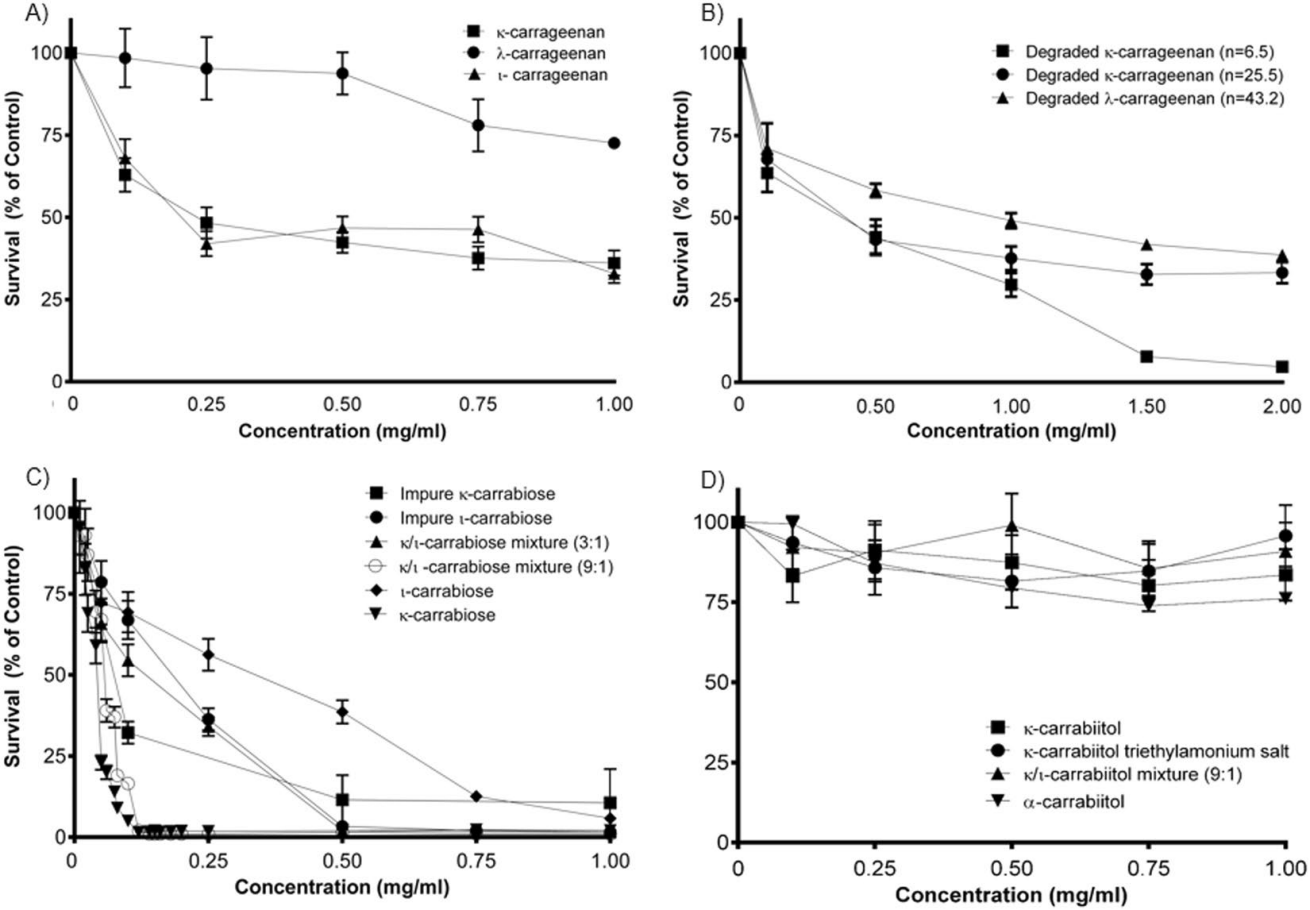
Cytotoxicity of carrageenan polysaccharides (**A**), oligosaccharides (**B**), carrabioses (**C**) and carrabiitols (**D**) in LM2 cells. LM2 cells were exposed to the compounds at different concentrations, during 48 h and afterwards, cell viability was quantified by the MTT assay. Cell death is referred as percentage of the vehicle treated control.

The first step was to evaluate the native κ-, ι- and λ-carrageenans. Figure 3A shows that κ- and ι-carrageenan have similar cytotoxic capacity, with IC_50_ values of 0.23 ± 0.02 and 0.20 ± 0.04 mg/ml respectively, inducing significant reduction of cell death respect to the control from 0.1 mg/ml onwards (p < 0.01, ANOVA, Dunnet post hoc test). On the other hand, λ-carrageenan only showed significant toxicity at 1 mg/ml. The presence of a higher number of sulfate groups in the disaccharidic unit appears to be correlated with a neglected cytotoxicity.

Certain cytotoxicity of compounds κ- and λ-carrageenans on Hela cells has already been reported, showing IC_50_ values of 0.55 and 0.47 mg/ml respectively after 72 h exposure^14^. We obtained a lower IC_50_ (0.23 mg/ml) upon 48 h incubation with κ-carrageenan but we did not find cytotoxicity at all for λ-carrageenan. This last result is consistent with the findings of Zhou *et al*.^12^, as they observed that λ-carrageenan is active *in vivo*, but not *in vitro*. Furthermore, other authors reported scarce cytotoxicity of undegraded carrageenans^18,21^. In the present work we have found, that besides κ-carrageenan, ι-carrageenan is also cytotoxic.

The differences observed between our results and those obtained by other authors may be based on the differences in terms of different susceptibilities of a given cancer type for any particular drug^35^, time exposure and serum percentage employed.

Li *et al*.^15^ reported that λ-carrageenan might act as an antitumor agent by modulating the immune response, i.e. an indirect effect of cell killing, promoting dendritic cells maturation and thus they proposed its use as an adjuvant of dendritic cell -based vaccines.

To the best of our knowledge, there are no previous reports of ι-carrageenan as a potential antitumor compound. Ariffin *et al*.^21^ studied the effect of κ- and ι-carrageenans in normal and cancerous intestine and liver human cell lines, and they did not show cytotoxicity effect in their experimental system.

Regarding the degradation products of carrageenans, Jin *et al*.^20^ demonstrated the *in vitro* antitumor activity of degraded ι-carrageenan against an osteosarcoma cell line, but ours is the first report of potential antitumoral activity of a non-degraded ι-carrageenan.

We could hypothesize a possible relationship between the structure of the compounds and their cytotoxicity, being the most active compounds κ- and ι-carrageenan, which contain less sulfate groups, thus suggesting that an abundance of these groups could contribute to lower its cytotoxic action. This is not surprising, since the presence of anionic ions would be lowering the interactions between the carrageenan and the cells, considering that membranes bear negative charges.

#### Oligosaccharides

In a second step, we hydrolyzed partially a cytotoxic (κ-carrageenan) and a non-cytotoxic (λ-carrageenan) polysaccharide, thus generating chemically characterized oligosaccharides that were evaluated under the same conditions. Figure 3B shows that the κ-oligosaccharides exert cytotoxicity within the same IC_50_ range as compared to the polysaccharides. Degraded κ-carrageenans with n = 25.5 and 6.5 show IC_50_ values of 0.39 ± 0.05 and 0.38 ± 0.04 mg/ml respectively, this toxicity being significantly higher than that found in for the undegraded κ-carrageenan (p < 0.01, ANOVA, followed by Tukey post hoc test), in spite of the reported fact indicating that degradation of the polysaccharides increase the cytotoxicity^12,21^.

On the other hand, the degraded λ-carrageenan (n = 43.2) presents a marked cytotoxicity (IC_50_ = 0.95 ± 0.11 mg/ml), much higher as compared to the parent polysaccharide (p < 0.01, ANOVA, followed by Tukey post hoc test), thus demonstrating that, in this case, partial hydrolysis of the polysaccharide improved its cytotoxic properties.

There are previous reports of the antitumor properties of degraded κ-carrageenans^18,21,36^ but to date there are no reports of cytotoxicity of a degraded λ-carrageenan *in vitro*.

#### Disaccharides and disaccharide-alditols

We have further continued the study with even smaller compounds, through modifications and purification (Fig. 1), which led to different disaccharides (carrabioses, Fig. 3C) or mixtures. The structures of the purified carrabioses, in particular, were confirmed by NMR spectroscopy (full assignment is shown in the Materials and Methods section). Data for κ-carrabiose is consistent with literature^37^, although to the best of our knowledge, data for ι-carrabiose has never been reported before. NMR data showed that carrabioses carry a masked aldehyde group in the form of a hydrate.

The carrabioses present a broad range of cytotoxicity values upon tumor cells. Figure 3C indicates that among the carrabioses, pure κ-carrabiose and the mixture 9:1 of κ- and ι-carrabiose are the most cytotoxic compounds (significantly higher than ι-carrabiose alone (p < 0.05, ANOVA, Tukey post hoc test), having IC_50_ values of 0.043 ± 0.009 mg/ml and 0.052 ± 0.005 mg/ml respectively, thus suggesting again the influence of the low sulfation degree on the toxicity of these compounds.

Among κ-carrabioses, the pure compound is more cytotoxic than the impure one, whereas among ι-carrabioses, the impure is more active (p < 0.05, ANOVA, Tukey post hoc test). IC_50_ values are as follows: 0.074 ± 0.009 mg/ml for impure κ-carrabiose, 0.183 for ± 0.015 mg/ml and 0.338 ± 0.035 mg/ml for impure and pure ι-carrabiose, respectively and 0.133 ± 0.012 mg/ml for the 3:1 mixture of κ- and ι-carrabiose.

These results indicate that depolymerization up to the disaccharide increases the cytotoxicity of the carbohydrates, at least for κ-carrageenan. It is also clear that κ-carrabiose shows a higher cytotoxicity than ι-carrabiose, suggesting that this capacity is not favored by sulfation on C-2 of the 3,6-anhydro sugar. As expected, the mixtures of of κ- and ι-carrabioses have intermediate IC_50_ values, suggesting that no synergistic effect between both disaccharides occur.

The presence of an aldehyde group (masked as a hydrate) in carrabioses generates a biological activity that is lost when these disaccharides are reduced to their alditols (carrabiitols), since the sulfate content is the same in both compounds. Both α- and κ-carrabiitol have also been analyzed by NMR spectroscopy (full assignment is shown in the Material and Methods section; data is consistent with literature^38^) and the purified products were tested for cytotoxicity. All the samples show no cytotoxicity at all (Fig. 3D), suggesting that the masked aldehyde group is key for keeping the cytotoxic activity.

It has been found that short chain aldehydes are hard electrophiles that cause toxicity, probably by forming adducts with hard biological nucleophile, e.g., primary nitrogen groups^39^ of proteins or nucleic acids.

It is worth to note that to the best of our knowledge, there are not any previous reports of the antitumoral activity of disaccharides derived from carrageenans identified and tested as antitumoral agents. Besides, κ-carrabiose has an IC_50_ 10–30 times lower than those of other oligosaccharides derived from carrageenans found in literature, even after longer exposition to the compounds^14,18,20^.

The IC_50_ of κ-carrabiose is near 100 μM, within the range of several antineoplasics commonly used tested under the same conditions of incubation time. Simon *et al*.^40^ established cell cultures from 30 mammary gland tumors excised from dogs and reported the following IC_50_ values for chemotherapeutics after incubation during 48 h: 0.34 μM for doxorubicin; 27.3 μM for cisplatin and 53.4 μM for carboplatin. Our results are highly encouraging, although further *in vivo* studies are needed to test the potential of this disaccharide as an antineoplasic agent. κ-Carrageenan should not be used in injectable form due to its anticoagulation properties^1,8^. On the other hand, κ-carrabiose, which does not have this property due to its low molecular weight, could be a potential agent for parenteral administration.

Purified κ-carrabiose was the compound exerting the highest cytotoxicity against LM2 tumor cells, and thus we selected this disaccharide for the following studies about its potential antitumor ability.

### Analysis of cytotoxicity of κ-carrabiose in different cell lines

The cytotoxicity of the most promising compound (i.e. κ-carrabiose) was additionally evaluated in a small panel of tumor cell lines of various origins such as ovary, blood and lung cancer, melanoma, squamous cell carcinoma, and leukemia. LM2 cells data is also included (Table 1). In the new cell lines evaluated, the obtained IC_50_ values ranging from 0.051 to 0.099 mg/ml, thus showing that κ-carrabiose is potentially cytotoxic in several tissues besides mammary.

**Table 1:** Cytotoxicity of κ-carrabiose in a panel of cell lines expressed as IC_50_ (mg/ml) after 48 h exposure in 5% serum-containing medium, Mean ± SD.

Cell line	CI_50_ (mg/ml)
LM2	0.043 ± 0.009
IGROV-1	0.099 ± 0.004
K562	0.049 ± 0.002
B16-F10	0.039 ± 0.005
MB49	0.045 ± 0.008
A549	0.066 ± 0.005
Pam212	0.051 ± 0.006

### Effect of purified κ-carrabiose on the LM2 cell cycle

**Figure 4:**
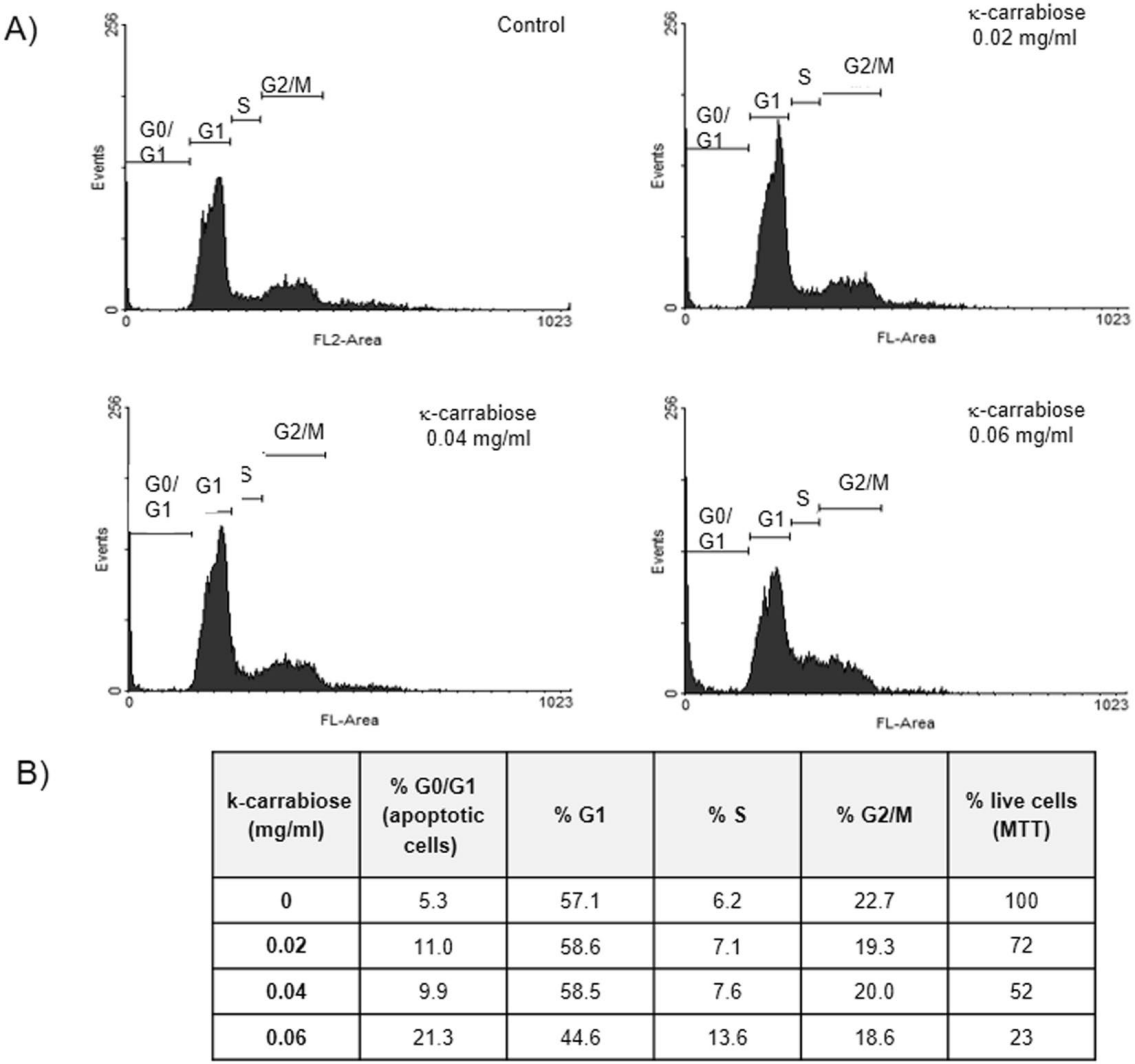
Influence of κ-carrabiose on LM2 cell cycle. LM2 cells were incubated with κ-carrabiose, at different concentrations, during 48 h and afterwards, the DNA was stained with propidium iodide and cell cycle was analyzed by flow cytometry as described in Materials & Methods. (**A**) The histograms show the number of cells (events) in each phase of the cell cycle. FL-area denotes propidium iodide fluorescence; (**B**) the Table shows the percentage of cells in each phase of the cycle and the correlation with the number of living cells determined by the MTT assay.

Figure 4 shows the analysis of cell cycle after propidium iodide staining of DNA of LM2 cells treated with purified κ-carrabiose. At concentrations higher than the IC_50_, such as 0.06 mg/ml, we observed an increase in the sub-G1 cell population (5.3%, control, vs. 21.3%), thus showing an increase in apoptotic cell death.

In addition, LM2 cells exposed to κ-carrabiose employed at 0.06 mg/ml, showed an increased arrest in S phase (6.2 to 13.6%) and a corresponding decrease of cells in the G_1_ and G_2_-M phases as compared to the control.

Cell death is typically discussed dichotomously as either apoptosis or necrosis. Apoptosis is described as an active, programmed process of autonomous cellular dismantling that avoids eliciting inflammation. Necrosis has been characterized as passive, accidental cell death resulting from environmental perturbations with uncontrolled release of inflammatory cellular contents. As apoptosis is considered to be a regulated and controlled process^41^, it is desirable that new compounds are able to elicit cell death via apoptosis^42^.

Most antineoplasics such as 5-fluorouracil, methotrexate, docetaxel, hydroxyurea etc, exert their action through apoptotic death of target cells as well as arrest in S phase^43–45^, similarly to the mechanism driven by the purified κ-carrabiose.

### Cellular mechanism of κ-carrabiose uptake

Mammalian cells are generally considered to be unable to employ polysaccharides for cell growth because the phospholipid bilayer in the cell membrane has very low permeability to sugars. This is not surprising, since there is only one known animal disaccharide sucrose transporter that was recently reported^46^. With the aim of determining if κ-carrabiose was being incorporated into the cells, we studied the influence of temperature on the uptake period of the disaccharide. Passive transport processes are typically assessed in a parallel incubation at 4 °C to disable any active transport proteins^47^. For that purpose, we exposed LM2 confluent cells to the maximum possible κ-carrabiose concentration (2 mg/ml). Under these conditions, no differential cytotoxicity was observed, thus suggesting that κ-carrabiose is not being incorporated into the cells at all or either being transported by passive diffusion, which is an unlikely mechanism possible for a disaccharide.

### Effects of purified κ-carrabiose on cell-cell and cell-substrate adhesion of LM2 cells

**Figure 5:**
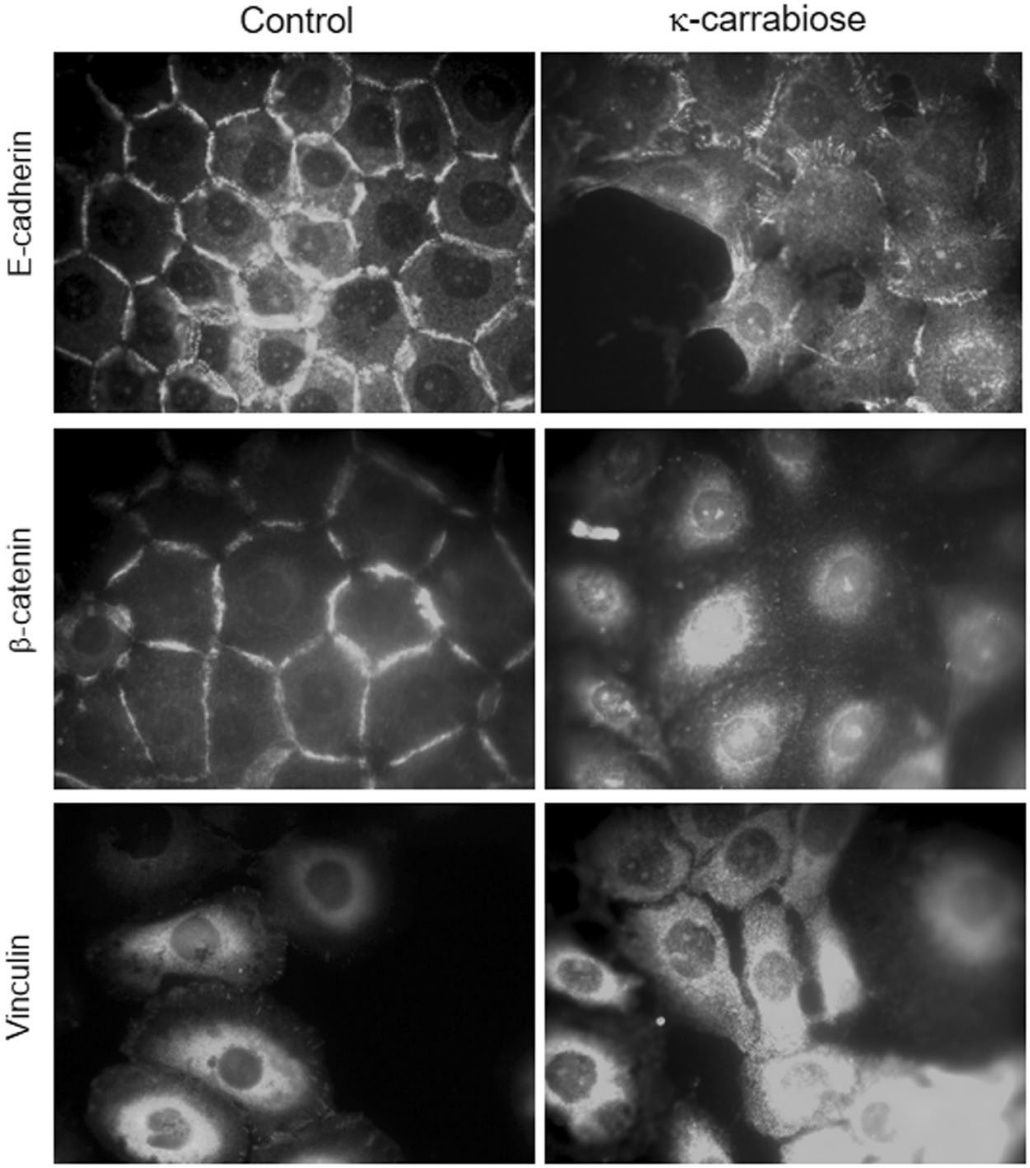
Effects of κ-carrabiose on cell-cell and cell-substrate adhesion of LM2 cells. Exemplary photographs of E-cadherin, β-catenin and vinculin immunofluorescence staining (detailed in Materials and Methods) after 48 h exposure to κ-carrabiose. Control cells exposed to vehicle were included. 100X magnification.

Figure 5 shows the impact of κ-carrabiose on the intracellular distribution of the cell-cell adhesion proteins E-cadherin and β-catenin and the cell-substrate adhesion protein vinculin. While LM2 control cells exhibited a normal E-cadherin distribution at the cell-to-cell interface, this distribution was disrupted in cells treated with κ-carrabiose and its expression resulted aberrant, with interdigitations appearing along the cell-cell interface together with a diffuse cytoplasmic signal. This pattern of distribution, accounts for higher cell–cell cohesion suggests and a higher cell differentiation level and thus a decrease in tumorigenesis^48^.

Similarly, β-catenin staining suggests an aberrant distribution after κ-carrabiose treatment. Whereas localization in the cell-cell contact areas is absent, a re-localization in the nucleus is present.

Two main groups of adhesion molecules: cadherins and cell-matrix adhesion molecules, have been implicated in tumor malignancy. However, the specific role that these proteins play in the context of tumor progression remains to be elucidated^49^. Beta-catenin was first identified at adherens junctions where it links cadherins with the cytoskeleton to regulate the response to cell adhesion. In addition, a small yet dynamic pool of β-catenin shuttles rapidly between the cytoplasm and nucleus^50^ and is responsible for transducing signals from plasma membrane to the nucleus, being an important step in cancer progression^51^.

In addition, vinculin distribution was confined to the focal adhesion points in LM2 control cells, whereas in κ-carrabiose treated cells a diffuse cytoplasmic pattern was observed. Vinculin provides a mechanical link controls cell signalling processes, affects contractility and adhesion protein turnover. Vinculin has been suggested to function as a tumour suppressor by supporting anchorage-dependent cell growth and by suppressing tumour metastasis through reducing cell motility, but this depends on the dimensionality, adhesiveness or steric hindrance of the environment^52^. However, as has previously found by our group^48^, vinculin expression in the focal contacts of the LM2 treated cells appears not to have a positive impact on cell migration.

In summary, κ-carrabiose affects cell–cell and cell-substrate adhesion of LM2 tumor cells through the topographic reorganization of some cytoskeletal and adhesion proteins, and these changes may be related to a differential cell migration observed *in vitro* in a wound assay.

The *in vivo* antitumor activity of carrageenans reported elsewhere^10^ has been hypothesized to be related to the destabilization of the interaction of the GAGs portion of the proteoclycans and the extracellular matrix proteins^11^. However, there are no previous reports on carrageenans or its derived disaccharides action on cell-to-cell interactions or on cell-substrate or extracellular matrix adhesion. However, normal adhesion process related to vinculin in B16 tumoral cells was altered when were seeded on sulfonated-chitosan films, which could reflect a possible *in vivo* delay on metastatic cell adhesion^53^.

### Effect of purified κ-carrabiose on the migratory ability of LM2 cells

Tumor metastasis is the most common cause of death in cancer patients. In carcinomas, the metastatic process is thought to consist of a number of distinct steps, invasion, intravasation, extravasation and proliferation. In the first step cells lose cell-cell adhesion and gain motility, which enables them to invade the adjacent tissue^54^.

**Figure 6:**
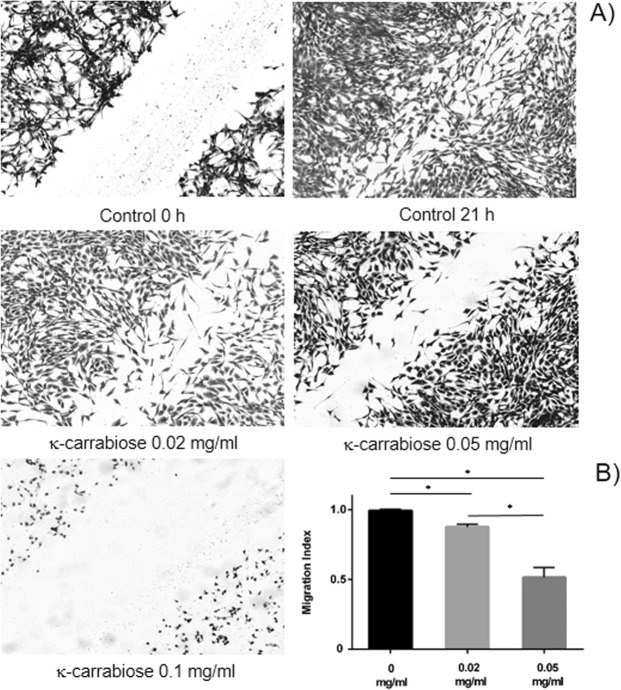
Wound healing assay of LM2 incubated with κ-carrabiose. Confluent LM2 monolayers were gently wounded and incubated in the presence of concentrations of κ-carrabiose. (**A**) After overnight incubation in presence of the compound (21 h) microscope photographs were taken. Controls of time 0 were carried out immediately after wounding; (**B**) Quantification of cell motility was carried out by measuring the distance between the invading fronts of cells in six random selected microscopic fields and the degree of motility was expressed as the Migration Index as detailed in Materials and methods. *p < 0.05, 1-way ANOVA test followed by Tukey post hoc test.

The first step of the metastatic cascade is the ability of cells to migrate and reach a blood vessel to intravasate and invade distant organs. Therefore, we tested the ability of LM2 tumor cells treated with purified κ-carrabiose to migrate and close an experimental wound (Fig. 6). It was shown that the strong migratory capacity of the non-treated cells was dramatically impaired in the cells pretreated with purified κ-carrabiose in all the concentration range assayed. In addition, a correlation between the the migratory decrease and the concentration of purified κ-carrabiose was also noticed. At 0.05 mg/ml, migration index was equal to: 0.52 ± 0.07. These results suggest that the cells surviving κ-carrabiose treatment would have a reduced ability to colonize distant organs, which is also important in terms of an effective anticancer treatment.

The highest concentration employed, that is 0.1 mg/ml, was not considered in the analysis since the cytotoxic effect, which is clearly observed in the Figure, does not allow us to evaluate the migratory ability.

## Conclusions

**Figure 7:**
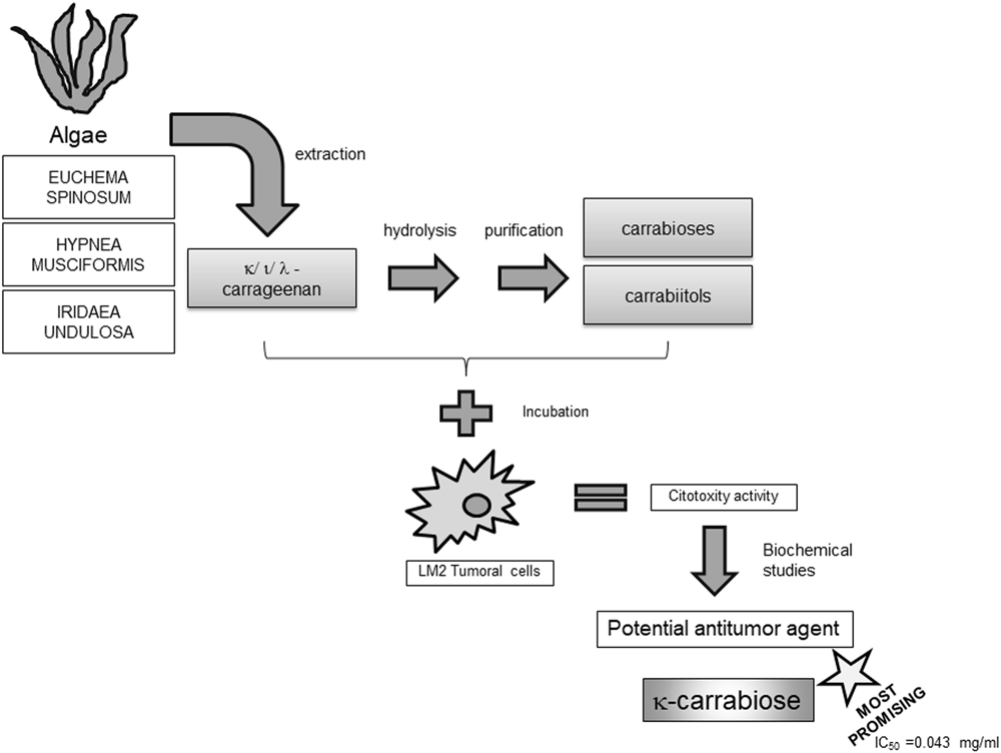
Summarized scheme of the results.

In this novel study of carrageenans and their derived oligosaccharides (see Fig. 7), we found that the presence of sulfate groups is a factor abrogating its potential cytotoxicity of native carrageenans, thus being the κ- and ι-carrageenans the more active agents among the polysaccharides.

In addition, some oligosaccharides are more cytotoxic than their parent compounds, thus showing that the lower molecular weight is one of the factors that improves its cell death killing ability. We have also focused on the study of the disaccharides obtained as degradation products of the carrageenans and their reduction products. None of the carrabiitols tested showed any significant cytotoxicity. Carrabioses, on the contrary, show the highest cytotoxic responses against tumor cells, being pure κ-carrabiose the most active of all the tested compounds. and thus we hypothesize that the aldehyde group could be the responsible for its biological activity.

We showed that this compound induces cell death through induction of G2/M phase arrest and apoptosis, changes on cell-cell and cell-matrix interactions probably exerting its action from outside the cells and thus potentially impairing the metastasizing ability of the surviving LM2 cells, suggesting a potential as an antitumor agent.

Potential applications of the results emerging from the present work include the use of disaccharide units such as carrabioses coupled to antineoplasics in order to improve its cytotoxicity and antimetastatic properties, and the use of ι-carrageenan as adjuvant or carrier in anticancer treatments. However, further studies extrapolating these results *in vivo* experiments are needed to assess carrabioses (and especially κ-carrabiose) as potential antitumor agents.
